# Postural changes and motor evoked potentials following percutaneous spinal cord epidural stimulation in spinal cord injury

**DOI:** 10.1038/s41394-026-00753-1

**Published:** 2026-09-11

**Authors:** Sohale Hessavi, Will Schroeder, Ahmad Alazzam, Muhammad Uzair Rehman, Robert Trainer, Ashraf S. Gorgey

**Affiliations:** 1 School of Medicine, Virginia Commonwealth University, Richmond, VA 23284 USA; 2 Spinal Cord Injury and Disorders Center, Richmond VA Medical Center, Richmond, VA 23249 USA; 3 Department of Biomedical Engineering, Virginia Commonwealth University, Richmond, VA 23284 USA; 4 Physical Medicine and Rehabilitation, Richmond VA Medical Center, Richmond, VA 23249 USA; 5 Department of Physical Medicine and Rehabilitation, Virginia Commonwealth University, Richmond, VA 23284 USA

**Keywords:** Translational research, Spinal cord diseases

## Abstract

**Introduction:**

Percutaneous spinal cord epidural stimulation (pSCES) is an effective modality for managing pain. However, the use of pSCES in restoration of motor control after SCI is still under research exploration. Today, the effects of postural changes on pSCES-motor control are still controversial.

**Case presentation:**

Two participants with complete SCI underwent implantation of pSCES. The motor recruitment curves of motor units of two different agonist-antagonist muscle groups were captured in a supine position and in a standing position using a standing frame. The amplitudes of the motor evoked response were presented in absolute or relative to the maximum response. Both narrow and wide field pSCES configurations elicited intensity-dependent EMG responses across lower-limb muscles, with substantially greater amplitudes in supine compared to standing. Transitioning to standing produced marked bilateral attenuation of raw responses across muscles and configurations, although relative recruitment patterns were partially preserved following EMG normalization. Increasing pulse duration broadened relative muscle recruitment and enhanced distal muscle activation, but posture remained the dominant determinant of overall response magnitude.

**Discussion:**

Standing posture was associated with substantial attenuation of pSCES-evoked responses across muscles and stimulation parameters, indicating posture-dependent modulation of evoked muscle activation following pSCES implantation in persons with SCI.

Spinal cord is a pivotal conduit of the central nervous system that regularly coordinates environmental demands or stressors with functional outcomes. These outcomes may vary based on the external needs to achieve sensory, motor or autonomic homeostasis [1]. Following spinal cord injury (SCI), such delicate coordination between the demands and the outcomes is disrupted and associated with loss in mobility, diminished sensorimotor control and impaired vasomotor response [2, 3]. More invasive percutaneous spinal cord epidural stimulation (pSCES) and less invasive transcutaneous spinal cord stimulation (tSCS) have emerged as potential rehabilitation interventions that possibly may restore spinal cord coordination relative to environmental demands [4–6]. Prior literature has shown that tSCS can activate and modulate sensorimotor networks similar to epidural stimulation to facilitate standing and stepping in patients with SCI [7–11]. Similarly, several studies have demonstrated that pSCES can improve sensorimotor control, restore the ability to stand and step independently, and enhance vasomotor responses to orthostatic challenge. [4, 5, 12, 13] The effectiveness of pSCES relies primarily on targeted delivery of electrical pulses to activate specific motor units and neural networks necessary to achieve functional outcomes [4, 5, 12, 13].

Spinal mapping becomes a key strategy to unravel the specific motor neurons and corresponding neural networks necessary to enable specific motor behaviors [10–13]. Spinal mapping is typically performed in a supine position and is accompanied by simultaneous EMG recordings from lower-extremity muscles [11, 12]. Once typical muscle-specific recruitment curve is established in an amplitude dependent manner, the optimum pSCES stimulation parameters and configurations are then considered [7, 11, 12]. Previously, peak slope ratio was used as a criterion method to filter different recruitment curves in response to different pulse durations (250, 500 and 1000 µs) and different cathodal-anodal configurations to achieve standing posture after SCI [12]. The findings noted that using the slope ratio of the agonist-antagonist muscles is likely to recognize the optimum stimulation parameters and configurations than reliance on visual observation of the induced motor evoked potentials (MEPs). The selected configurations should be then re-examined in a standing position to optimize the stimulation parameters (frequency, pulse duration and amplitude) necessary to achieve standing [12]. The goal is to identify stimulation parameters that maintain consistent neural activation across postural conditions. Prior work using lumbar tSCS has demonstrated that body position can influence neural recruitment and motor responses. Danner et al. reported that changes in body position altered the neural structures recruited by lumbar tSCS, highlighting that stimulation responses are not fixed properties of electrode configuration alone but are also shaped by posture-dependent changes in spinal and peripheral biomechanics [14]. However, although these findings provide important context for posture-dependent modulation during non-invasive stimulation, the extent to which similar posture-related attenuation occurs during pSCES mapping remains less well characterized. Therefore, it remains unclear how postural transitions from lying to standing influence recruitment of targeted motor units during pSCES mapping in persons with SCI.

Postural changes influence the distance between pSCES electrodes and the spinal cord neural circuitries [15]. Neural activation during pSCES may be further attenuated by the circulating cerebrospinal fluid (CSF), which typically has a dorsal thickness of approximately 2.0–3.2 mm after altering position from supine to standing [16–18]. The increase in CSF thickness resulted in fewer axon-neural circuitries being activated especially with constant amplitude [19]. Such effects may limit the ability of pSCES to effectively restore motor control required for functional tasks such as standing. Previous trials demonstrated that persons with complete and motor complete SCI effectively achieved sitting trunk control and standing following implantation of percutaneous pSCES [5, 12]. However, these trials did not quantify the magnitude of posture-related changes in pSCES-evoked motor responses in target muscles. Therefore, the purpose of the current case report is to examine the effects of postural changes (supine to standing) on pSCES-MEP in response to either narrow or wide field configurations and after altering pulse durations (250, 500 and 1000 µs) in two persons with motor complete SCI.

## Materials and methods

### Subjects

Two men with chronic, traumatic, clinically motor complete SCI (C8; 6 years post-injury [ID#: 0881], T11; 9 years post-injury [ID#: 0882]), American Spinal Injury Association Impairment Scale [AIS] A and B, respectively participated in a clinical trial approved by the Richmond Veteran Affairs (VA) Medical Center [22]. The neurological level of injury was determined using the ASIA/ISCoS International Standards for Neurological Classification of Spinal Cord Injury (ISNCSCI). Participants 0881 [25 mcg of cholecalciferol daily and 283 mg of docusate daily] and 0882 [100 mg of sildenafil citrate, once daily and 100 mg Dilantin ER, 4x daily] were on the listed medications during the course of the trial.

Participants were recruited through the VA Spinal Cord Dysfunction Registry and provided written informed consent after receiving detailed explanations of the study objectives, surgical implantation of percutaneous epidural electrodes, stimulation procedures, and the use of their data for research and publication. The clinical trial was registered at clinicaltrials.gov on April 3, 2021 (NCT04782947) [22].

### Timeline of study

The two participants included in the present analysis (ID# 0881 and 0882) were enrolled in a longitudinal clinical trial with an overall duration of approximately 12 months. The parent study investigated the effects of resistance training (RT) combined with percutaneous spinal cord epidural stimulation (pSCES; REST SCI) on restoring standing performance and overground locomotion in individuals with chronic SCI [4, 20]. The intervention period was divided into two primary phases. The initial 24 weeks focused on improving standing capacity, followed by an additional 24 weeks directed towards restoration of overground locomotion. Throughout the intervention, participants trained (3x per week) using exoskeleton-assisted walking (EAW) for approximately 1 h per session, followed by an additional 1 h of standing re-training (task-specific training). Participants were randomized into either pSCES + EAW + RT or delayed pSCES + EAW + no RT groups. Both participants (0881 & 0882) were randomized to the immediate intervention group (pSCES + EAW + RT). Clinical and functional assessments were performed at baseline (prior to training), following completion of the first 24-week phase (post-measurement 1), and at the end of the second 24-week phase (post-measurement 2). The present case report represents an initial analysis from the ongoing parent trial. These two participants were included because they underwent pSCES implantation and posture-specific spinal mapping while the mapping protocol was being developed and refined. Because pSCES implantation and posture-specific spinal mapping are technically demanding, resource-intensive and require substantial participant time, clinical coordination and specialized personnel, the initial analysis was intentionally limited to a small sample to assess feasibility and characterize preliminary recruitment patterns. Additionally, posture-specific pSCES mapping was not the primary efficacy endpoint of the parent trial; therefore, the present analysis was designed to characterize preliminary posture-dependent motor recruitment patterns rather than to test generalizable clinical efficacy. Detailed inclusion and exclusion criteria have been previously reported [4, 12, 20]. Briefly, eligible participants were adults aged 18–60 years with chronic (≥24 months post-injury), traumatic, motor complete SCI below the C5 neurological level. All participants underwent International Standards for Neurological Classification of SCI (ISNCSCI) examinations to determine neurological level and severity of injury, and only individuals classified as AIS A or B were included to facilitate detection of potential changes in voluntary motor control below the level of injury.

### Implantation of percutaneous SCES

Participants underwent a two-step implantation procedure in which temporary pSCES implantation preceded permanent implantation. The pSCES system was delivered using the Intellis^TM^ Epidural Stimulation System (Medtronic, Minneapolis, MN, USA) to electrically stimulate the lumbosacral spinal circuitry. Surgical techniques for both temporary and permanent implantation have been previously reported [4, 20].

For temporary implantation, participants provided written informed consent and were positioned prone on a fluoroscopy table in a sterile operating environment. A time out was held for patient verification. The skin was prepped and draped in a normal sterile fashion. Standard intraoperative monitoring was used throughout the procedure. A nurse certified in sedation established IV access, placed standard ASA monitors (Philips Medical Systems, BG Eindhoven, The Netherlands) including noninvasive blood pressure every 5 min, pulse oximetry, continuous EKG, and end tidal CO_2_ monitoring via a nasal cannula. The participant received 2 g of Ancef prior to the procedure. Each participant was adequately sedated throughout the procedure to allow participant interaction with the surgeon while minimizing discomfort, anxiety, or distress. Using anteroposterior (AP) and lateral fluoroscopic guidance, the L3 vertebral body was identified, and local anesthesia was applied. One percent (1%) lidocaine was used for local skin infiltration of the tract and the lamina of L3. Next two 14-gauge Medtronic 6-inch needles were guided to the interlaminar space between L2 and L3 vertebral bodies at an angle of approximately 30 degrees using a loss-of-resistance technique through use of a glass syringe with normal saline and air. Afterwards, the stylette was removed and two Medtronic leads were placed through each of the 2 needles from the right of the midline and left of the midline and advanced into the epidural space under live AP fluoroscopy until the rostral end of each lead was positioned near the superior aspect of T11 and the 8 contacts of each percutaneous lead spanned the spine to the L1 vertebral body covering the area of the conus medullaris [4, 20].

A Medtronic representative interrogated the lead to ensure normal epidural impedance, while posterior epidural space placement of the lead was confirmed through a lateral view under fluoroscopy. In addition, the location of leads was confirmed by visual inspection of evoked contractions in paralyzed lower extremity musculature during stimulation testing. The needles were then removed under live fluoroscopy to maintain the lead position, and final X-ray images were taken following application of Dermabond and Steri-Strips to anchor the leads to the skin. The area was covered with Op-Site and gauze with additional tape on top to provide more cutaneous wound stability. The Medtronic Bluetooth receiver was placed on the back laterally and confirmed communication with the interrogator [4, 12, 20].

Approximately 2–4 weeks following temporary implantation, two 8-electrode arrays of Vectris percutaneous leads were used for permanent implantation in an operating room under sterile conditions with anesthesiology support. The permanent procedure followed the same anatomical targeting and fluoroscopic guidance procedures as previously described for the temporary implantation procedure [4, 12, 20]. An IV line was established for sedation, and standard ASA monitors were placed including noninvasive blood pressure every 5 min, pulse oximetry, continuous EKG, and end tidal CO_2_ from a nasal cannula. Prophylactic antibiotics (cefazolin 2–3 g or clindamycin 600–900 mg) were administered at the time of implantation. Under fluoroscopic guidance, two separate 5-inch, 14-guage epidural needs were placed using the loss-of-resistance technique to access the epidural space. The leads were subsequently advanced within the epidural space, and stimulation configurations necessary to induce electrical activation of the targeted musculature were re-evaluated. A midline cutdown was performed to expose the leads and remove them from the needles under live fluoroscopy. A second incision was created to house the battery, and the leads were anchored to the surrounding fascia using non-absorbable sutures and tunneled subcutaneously to the pulse generator. Following hemostasis, wound incisions were closed in a layered fashion with sterile dressings applied i.e., (derma-bond, occlusive dressing, tape). Participants received short-term (3 day supply) postoperative analgesic medication, with recovery typically completed within 7–10 days. Following permanent implantation participants were instructed to avoid strenuous physical activity for 3–4 weeks to allow adequate lead stabilization. Post-implantation evaluations included routine wound checks, device interrogation, and spinal mapping sessions [4, 12, 20].

### Spinal pSCES segmental mapping

Following permanent implantation, participants underwent scheduled spinal segmental mapping procedures. pSCES mapping was performed to identify optimal cathodal-anodal electrode configurations and stimulation parameters, which included frequency, amplitude, and pulse duration to enable functional volitional movements, while minimizing unintended muscle activation as previously noted. Spinal mapping was conducted daily for 2 weeks immediately following permanent implantation and was repeated during an interim mapping phase conducted over 4 weeks following the first 24 weeks of the study. During mapping session, systematic efforts were made to determine the most effective cathodal and anodal combinations for specific muscle groups and joints, as well as appropriate stimulation frequencies (2–40 Hz) and pulse durations (250–1000 µs). Participants 0881 and 0882 underwent spinal mapping to generate recruitment curves during weeks 27–29 of the study. The lowest stimulation amplitude (mA) necessary to evoke visible muscle contraction was targeted (1–10 mA), with adjustments made as needed throughout the trial to monitor changes in neuromuscular responsiveness.

### EMG data analysis and recruitment curve development

Spinal mapping was performed in a supine position and then repeated in a standing posture using the same acquisition workflow. The standing posture was achieved using the large Easy Stand Evolv Glider sit to stand standing frame (Altimate Medical, Inc., Morton, MN, USA). Following transitioning from sit to standing, the standing frame was adjusted to provide support of both knees and hips to maintain upright posture. Once positioned, participants were instructed to remain relaxed to avoid voluntary lower-extremity movement while pSCES was delivered. Surface wireless EMG sensors were placed bilaterally over the targeted lower extremity muscles after shaving and cleaning the skin with alcohol swabs. Surface EMG activity was recorded for the key muscles, including the rectus femoris (RF), vastus medialis (VM), hamstrings (HS), tibialis anterior (TA), and medial gastrocnemius (MG). All EMG signals were collected at a 2000 Hz sampling rate using LabChart 8.1.21 (Windows; A.D. Instruments, Sydney, Australia). Individual channels were configured in LabChart for each muscle, recording was initiated, and baseline EMG activity was collected prior to stimulation. Stimulation was delivered using pre-identified electrode configurations and predefined stimulation programs, with pulse duration and frequency held constant within each program while stimulation amplitude was incremented across the planned range. For each stimulation amplitude, EMG was recorded continuously for 8 s, after which the next amplitude was applied until completion of the full amplitude series for that configuration. This sequence was repeated for each preset configuration, and all recordings were saved for offline processing. Supine recruitment curves were developed first and used to guide selection of the configurations advanced for evaluation in standing, prioritizing configurations that produced clear and segmentally appropriate responses in the target muscles.

For signal processing, EMG data were band-pass filtered (10–999 Hz) and rectified. Filtered, rectified signals were segmented into 0.4 s intervals across each 8 s recording window, and interval values were averaged to generate a single representative response value for each stimulation amplitude within each configuration. Recruitment curve development was performed separately for each muscle by compiling the averaged response values across all tested stimulation amplitudes for a given configuration. To facilitate comparison of postural related differences in muscle recruitment, responses were normalized to the single highest evoked response observed in the supine and standing position for each muscle group for each pSCES configuration and pulse width duration combination. The normalized values were then used to construct configuration-specific recruitment curves. The same signal processing and recruitment curve workflow was repeated for the standing mapping session.

To examine posture-related changes, supine and standing datasets were compared using both raw (non-normalized) and normalized responses for right- and left-sided muscles. The recruitment curves of different muscle groups were presented in supine and standing postures for both narrow central and wide field configurations as well as following adjustments to different pulse durations (250, 500, 1000 µs).

## Results

**Fig. 1:**
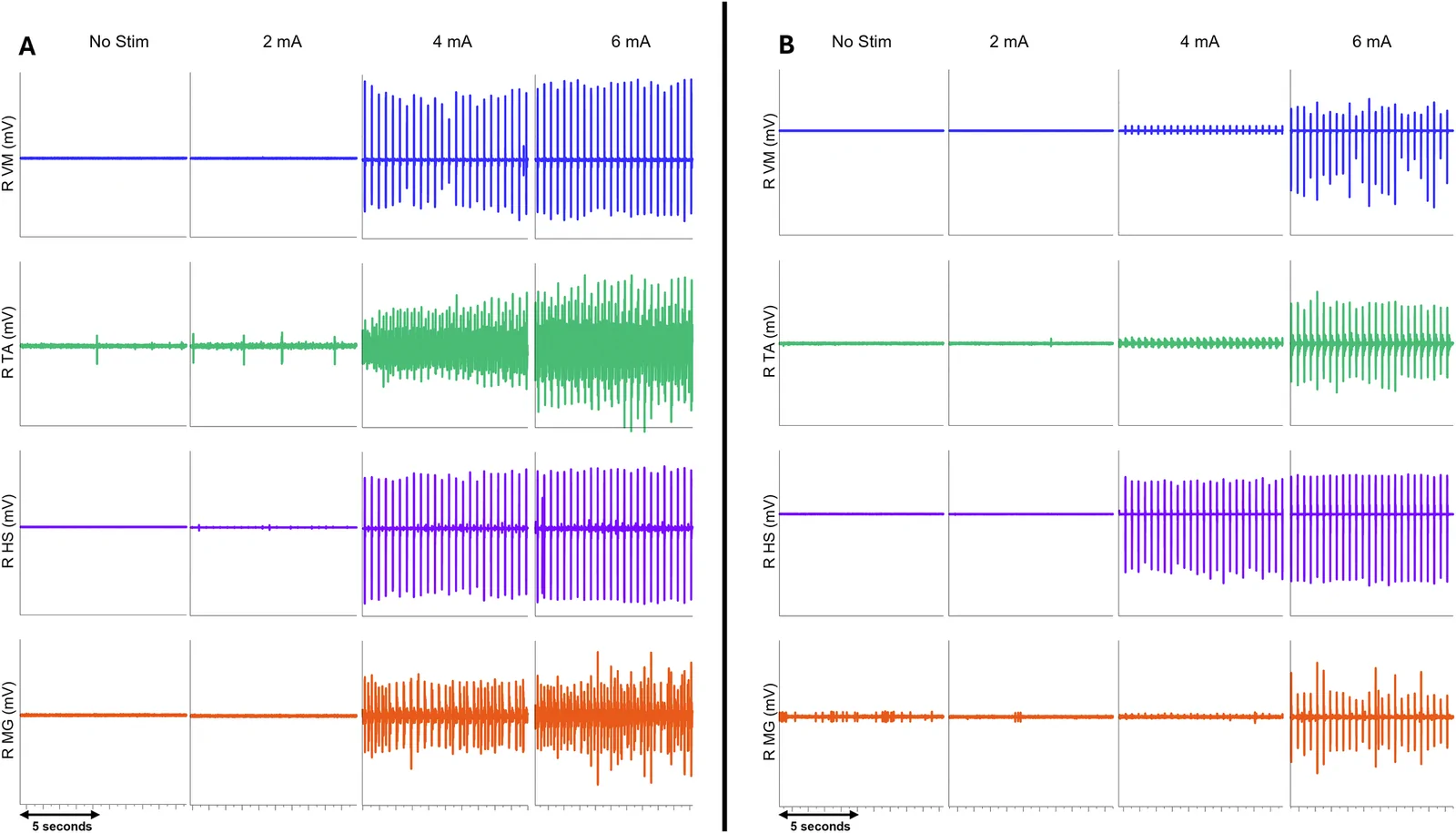
Representative lower extremity electromyographic recordings from Participant 0881 during percutaneous spinal cord epidural stimulation (pSCES) in the supine and standing postures. Surface electromyographic signals are shown from the right vastus medialis (R VM), tibialis anterior (R TA), hamstrings (R HS), and medial gastrocnemius (R MG). Recordings were obtained without stimulation and during stimulation at 2, 4, and 6 mA. Stimulation was delivered at 2 Hz with a 500-µs pulse duration using the +3/ + 11, −4/ − 12 electrode configuration. **A** Raw EMG signals recorded in the supine posture. **B** Raw EMG signals recorded in the standing posture. Each trace shows a representative 10 s recording epoch.

Figure 1 highlights representative lower extremity electromyographic recordings from Participant 0881 during percutaneous spinal cord epidural stimulation (pSCES) in the supine and standing postures.

### Narrow-central field configurations in 0881

**Fig. 2:**
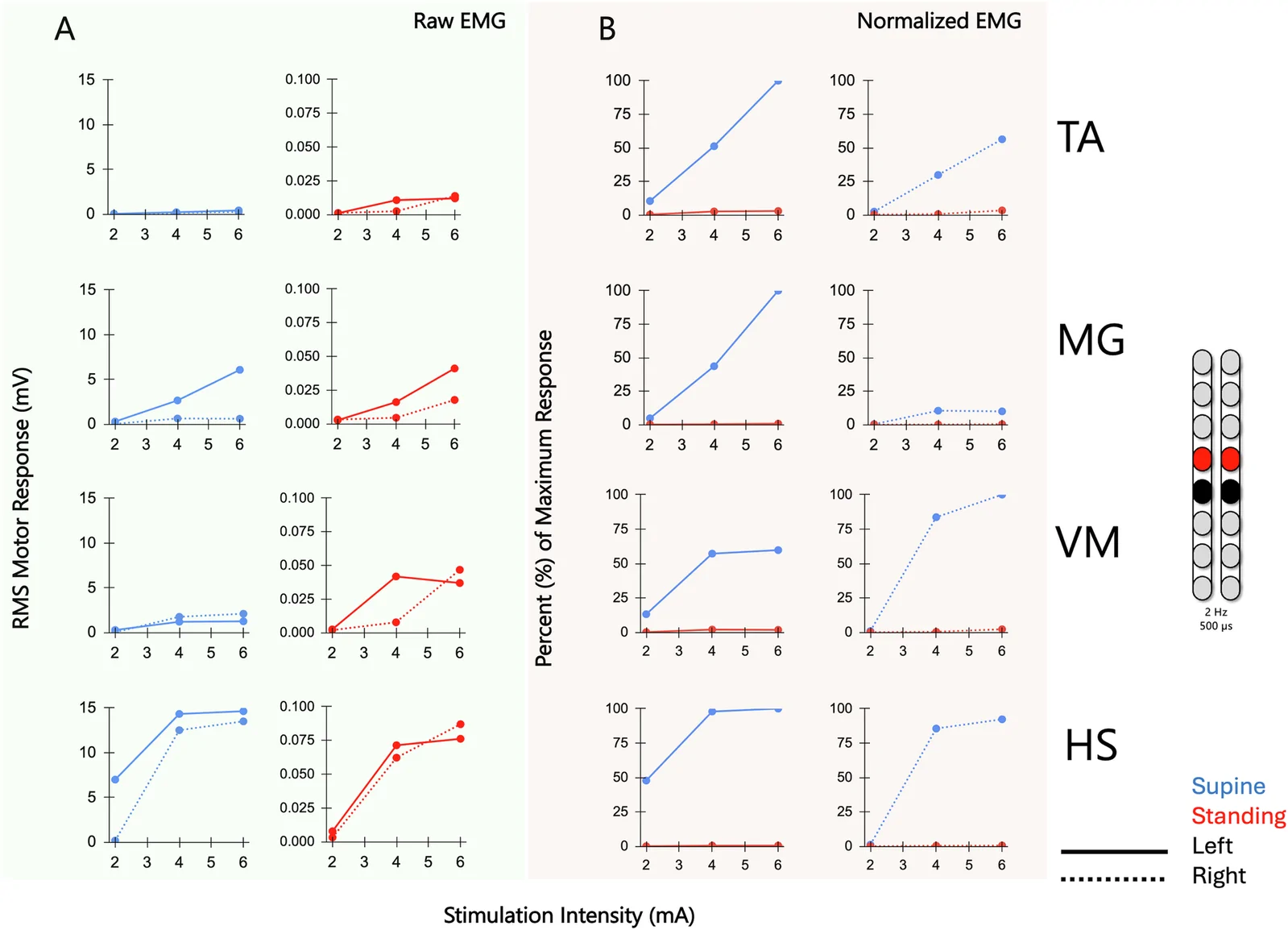
pSCES-evoked recruitment curves of lower-limb flexor and extensor muscle groups in supine and standing postures for participant 0881 after using central narrow field electrode configurations [+3, +11, −4, − 12]. Stimulation was delivered at 2 Hz with a 500 µs pulse width at current intensities of 2, 4, and 6 mA. **A** Raw EMG response amplitudes (mV) for bilateral tibialis anterior (TA), medial gastrocnemius (MG), vastus medialis (VM), and hamstrings (HS) in supine (blue) and standing (red) postures. **B** EMG responses normalized to the maximum response observed for each muscle across stimulation intensities. Solid lines denote left-sided muscles and dashed lines denote right-sided muscles.

Figure 2 displays the pSCES evoked recruitment curves following narrow central field electrode configurations [+3, +11, −4, −12] in participant #0881. The current was incrementally increased and was delivered at a pulse duration of 500 µs and a frequency of 2 Hz. Absolute raw EMG (Fig. 2A) demonstrated monotonically increases with stimulation intensity across all muscle groups, with consistently larger responses observed in the supine posture. Among the 4 muscle groups, HS exhibited the largest absolute responses, reaching 14.63 and 13.50 mV in the left and the right HS, respectively, during supine position, and 0.076 and 0.087 mV in the left and the right HS during standing posture, with comparable bilateral activation. Additionally, muscle activation in proximal muscle groups (VM and HS) plateaued after 4 mA stimulation intensity, whereas MG continued to increase with higher stimulation intensities.

Transitioning from supine to standing resulted in bilateral attenuation of evoked responses across all muscles, with standing responses consistently reduced relative to supine across different stimulation intensities and muscle groups, indicating posture-dependent modulation of evoked muscle activation. Despite the reduction in absolute amplitude, the overall shape of the recruitment curves was preserved across postures, with response amplitudes increasing monotonically with stimulation intensity in both conditions, indicating maintenance of relative intensity-dependent scaling of muscle activation between postures.

Normalization to each muscle’s maximal response across stimulation intensities (Fig. 2B) revealed muscle-specific posture-dependent differences in relative recruitment. The muscle maximal response occurred in supine lying position for all muscle groups. Proximal muscles (VM and HS) demonstrated rapid recruitment between 2 and 4 mA followed by early plateauing, particularly in the supine posture. In contrast, distal muscles demonstrated posture-dependent recruitment patterns, with MG exhibiting continued graded recruitment with increasing stimulation intensity in supine but limited recruitment in standing, whereas TA responses remained minimal across intensities in both postures. Collectively, these findings indicate that normalization accentuates posture-dependent differences in recruitment dynamics, with preferential proximal muscle plateauing and reduced distal muscle engagement during standing posture.

### Wide-field configurations in 0881

**Fig. 3:**
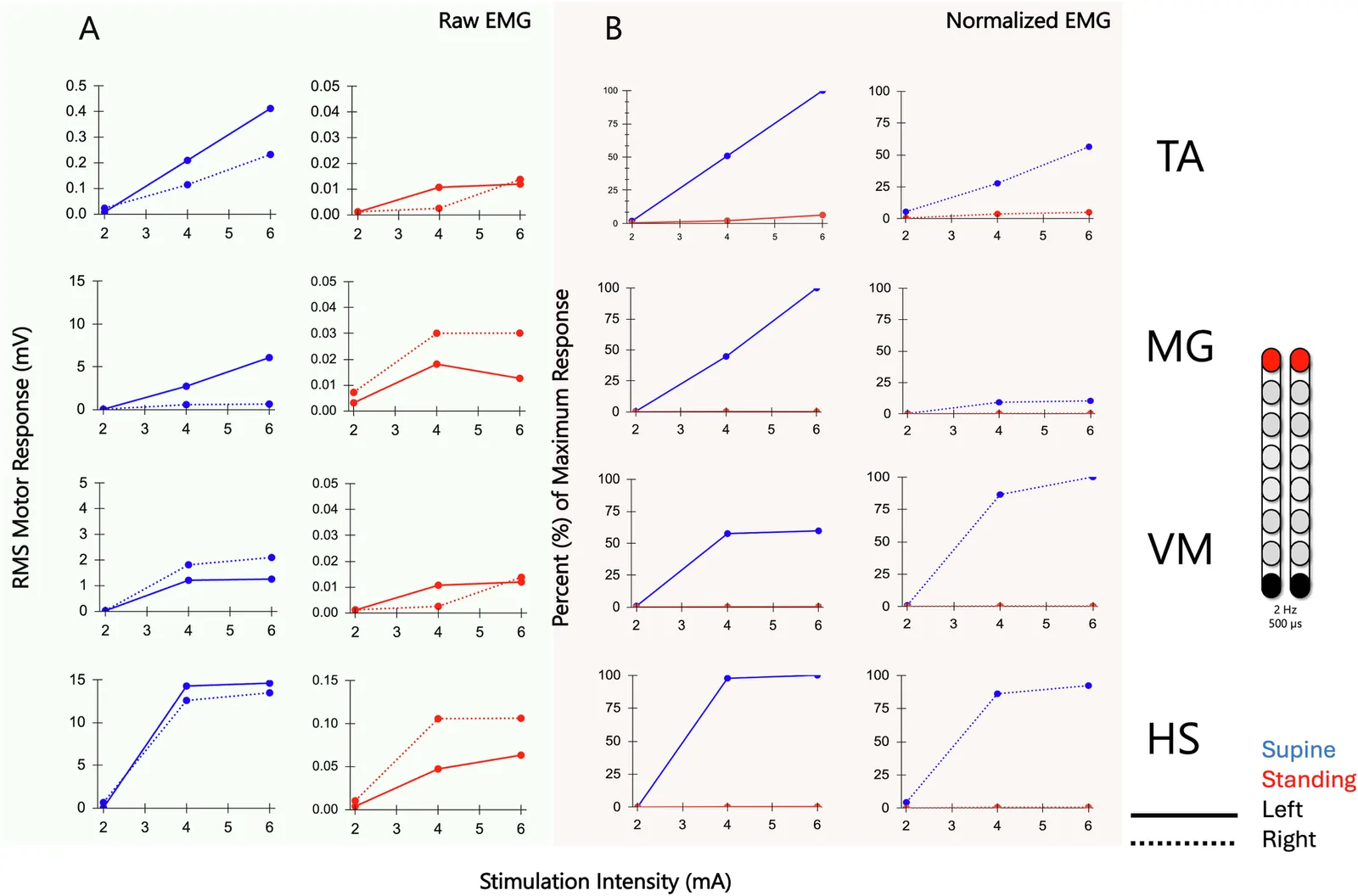
pSCES-evoked recruitment curves of lower-limb flexor and extensor muscle groups in supine and standing postures for participant 0881 using wide field electrode configurations [+0, +8, −7, −15]. Stimulation was delivered at 2 Hz with a 500 µs pulse width at current intensities of 2, 4, and 6 mA. **A** Raw EMG response amplitudes (mV) for bilateral tibialis anterior (TA), medial gastrocnemius (MG), vastus medialis (VM), and hamstrings (HS) in supine (blue) and standing (red) postures. **B** EMG responses normalized to the maximum response observed for each muscle across stimulation intensities.

Figure 3 highlights the pSCES evoked recruitment curves following wide-field electrode configurations [+0, +8, −7, −15] in participant # 0881. pSCES continued to evoke intensity-dependent EMG responses across lower-limb muscle groups in both postures (Fig. 3A). In the supine posture, absolute raw EMG amplitudes increased progressively with stimulation intensity across all muscle groups, with posterior muscle groups (HS and MG), exhibiting the largest response magnitudes. HS reached peak raw amplitudes of 14.63 mV (left) and 13.50 mV (right), whereas the left MG reached a peak amplitude of 6.07 mV at a stimulation intensity of 6 mA. In contrast, anterior muscles exhibited substantially lower maximal responses, with VM peaking at 1.25 mV in the left and 2.10 mV in the right, and TA peaking at 0.41 mV in the left and 0.23 mV in the right.

Transitioning from supine to standing produced a marked attenuation of raw motor evoked responses across all muscle groups, with reductions ranging from approximately tenfold to over one hundredfold in some muscles. Peak HS responses decreased from approximately 15 mV in supine to <0.1 mV during standing. Despite this attenuation, responses during standing remained intensity dependent across the tested stimulation range.

**Fig. 4:**
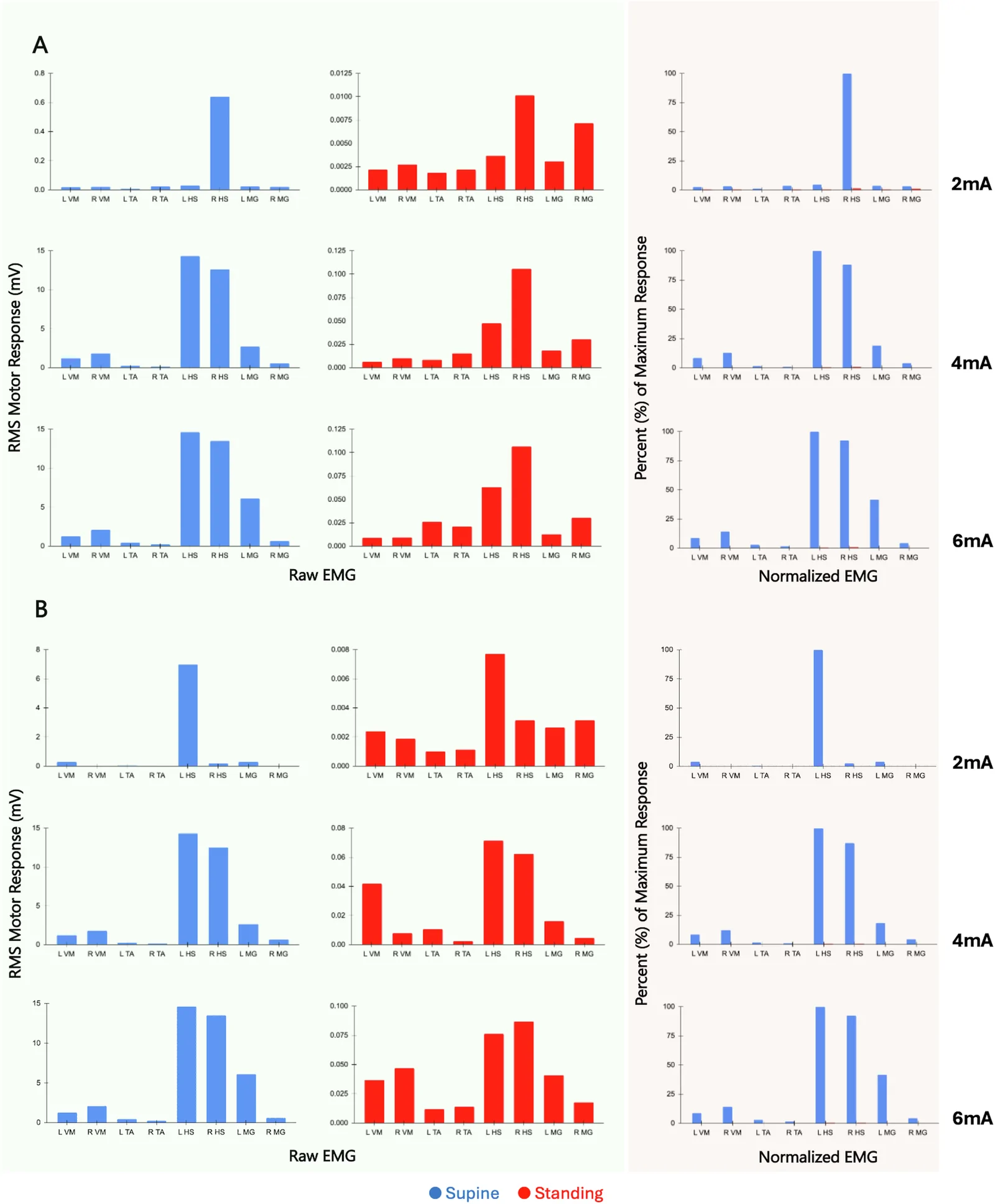
Configuration-dependent distribution of pSCES-evoked EMG responses in participant 0881. **A** Raw and normalized EMG responses recorded using electrode configuration [+0, +8, −7, −15]. **B** Raw and normalized EMG responses recorded using electrode configuration [+3, +11, −4, −12]. Stimulation was delivered at 2 Hz with a 500 µs pulse width at current intensities of 2, 4, and 6 mA. The same EMG data shown as recruitment curves in Figs. 1 and 2 were plotted as bar graphs showing bilateral peak-to-peak EMG amplitudes and normalized responses, expressed as a percentage of the maximum response observed for each muscle bilaterally across stimulation intensities within each configuration. Blue bars indicate responses recorded in the supine position, and red bars indicate responses recorded during standing. Tibialis anterior (TA), medial gastrocnemius (MG), vastus medialis (VM), and hamstrings (HS).

Normalization to each muscle’s maximal output (Fig. 3B) revealed marked posture and muscle specific recruitment patterns. In the supine posture, the left limb demonstrated robust intensity dependent scaling across muscles, with TA and MG approaching maximal activation by 6 mA, HS reaching near-maximal activation by 4 mA, and VM exhibiting a submaximal plateau at approximately 60% of maximum. The right limb displayed more heterogeneous recruitment, characterized by a gradual intensity-dependent increase in TA activation, minimal activation in MG, and strong recruitment of proximal muscles, with VM and HS approaching near-maximal activation at higher stimulation intensities (Fig. 4). In contrast, during standing, normalized responses were markedly attenuated bilaterally, with little to no observable recruitment across stimulation intensities in any muscle group. Together, these findings indicate that wide field stimulation preserves intensity dependent recruitment primarily in the supine posture, while standing is associated with near-complete suppression of normalized muscle activation across both limbs.

### Posture-dependent recruitment patterns and pulse durations

**Fig. 5:**
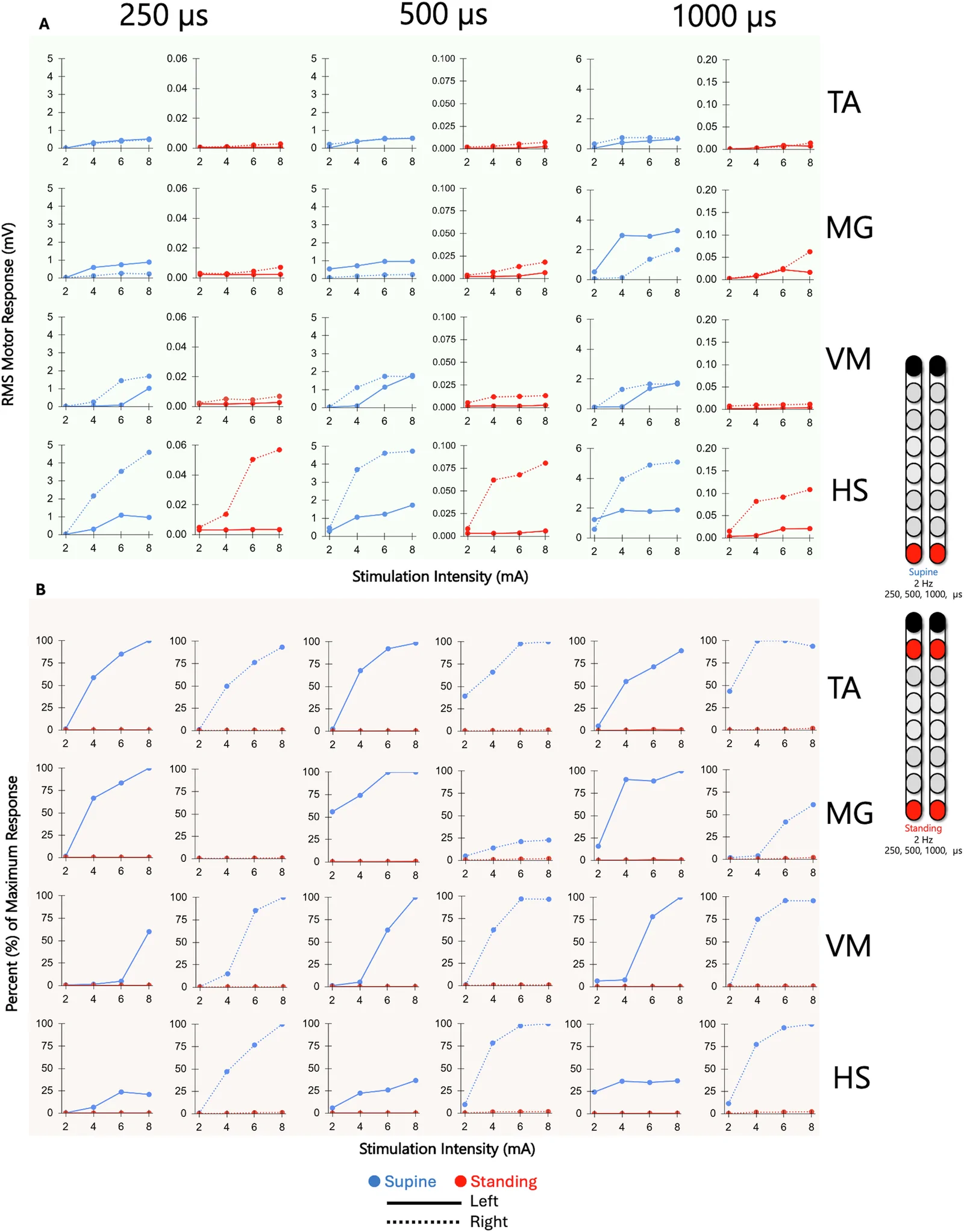
pSCES-evoked recruitment curves of lower-limb flexor and extensor muscle groups in supine and standing postures for participant 0882 using posture-specific electrode configurations. Raw and normalized EMG responses were recorded during transcutaneous epidural spinal cord stimulation using a supine (blue) configuration [+7, +15, −0, −8] and a standing (red) configuration [−0, −8, +1, +7, +9, +15]. **A** Raw EMG response amplitudes across left (solid line) and right (dashed line) tibialis anterior (TA), medial gastrocnemius (MG), vastus medialis (VM), and hamstrings (HS) muscle groups and (**B**) normalized EMG responses are shown across stimulation intensities at pulse widths of 250, 500, and 1000 µs.

The posture-dependent recruitment patterns were then examined at different pulse durations (250, 500, 1000 µs). We hypothesized that the posture-dependent recruitment pattern will be maintained independent of the adjusted pulse duration. pSCES-motor evoked responses were evaluated in participant 0882 using specific electrode configurations (supine: [+7, +15, −0, −8]; standing: [−0, −8, +1, +7, +9, +15]) (Fig. 5).

Raw EMG responses (Fig. 5A) increased with stimulation intensity across pulse widths and muscle groups, with consistently larger amplitudes observed in the supine posture. Posterior musculature, particularly HS, exhibited the greatest absolute activation across conditions, whereas TA and VM responses remained comparatively small. MG responses showed intermediate amplitudes that increased with stimulation intensity and pulse width. Overall response patterns were qualitatively similar across pulse widths and were consistent with the posture-dependent recruitment profile observed in participant 0881.

Transitioning from supine to standing produced bilateral attenuation of raw EMG amplitudes across muscle groups and pulse widths. Despite reduced absolute amplitudes during standing, intensity dependent scaling of responses remained evident across stimulation intensities for several muscles, indicating preservation of recruitment curve shape across postures.

**Fig. 6:**
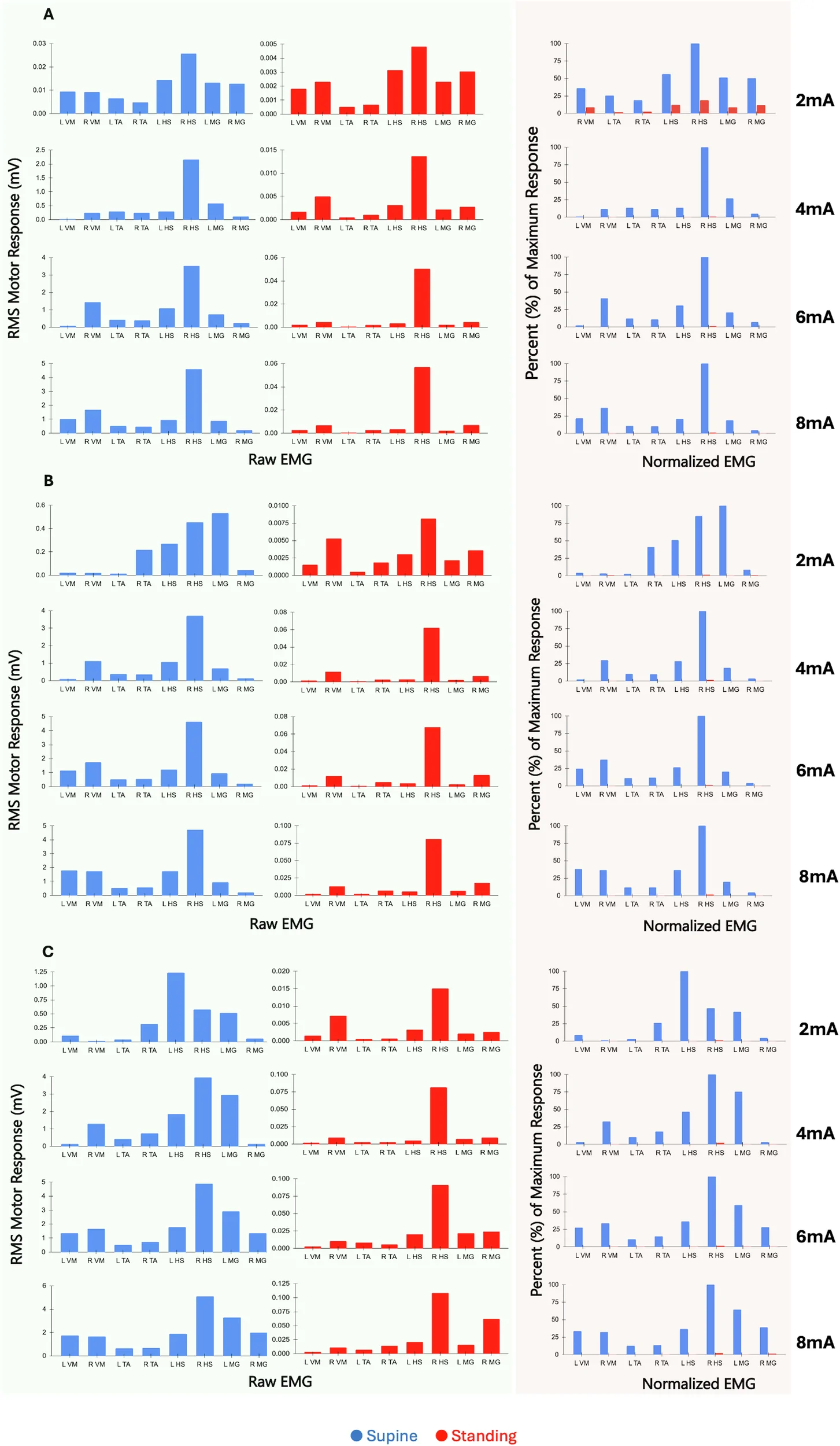
Pulse width–dependent distribution of pSCES-evoked EMG responses in participant 0882. Raw and normalized EMG responses recorded during spinal cord epidural stimulation using posture-specific electrode configurations (supine: [+7, +15, −0, −8]; standing: [−0, −8, +1, +7, +9, +15]) are shown as bilateral bar plots for pulse widths of (**A**) 250 µs, (**B**) 500 µs, and (**C**) 1000 µs. Stimulation was delivered at 2 Hz across current intensities of 2, 4, 6, and 8 mA. Normalized responses are expressed as a percentage of the maximum response observed for each muscle within each pulse width. Tibialis anterior (TA), medial gastrocnemius (MG), vastus medialis (VM), and hamstrings (HS).

Normalization to each muscle’s maximal response (Fig. 5B) revealed pulse width dependent differences in relative recruitment. At 250 and 500 µs, normalized activation was dominated by the supine posture across most muscles, with limited relative recruitment during standing. Increasing the pulse width to 1000 µs broadened relative recruitment and enhanced distal muscle engagement, most prominently in MG and TA (Fig. 6). Proximal muscles, including HS and VM, continued to reach high normalized activation in the supine posture across pulse widths (Fig. 6). Together, these findings indicate that increasing pulse width expands the distribution of relative muscle recruitment, while posture remains a primary determinant of overall activation magnitude.

## Discussion

The major findings of the current brief report are that comparison of raw and normalized SCES-evoked motor responses in two participants with complete (AIS A) and motor complete (AIS B) SCI revealed several key observations. The most prominent observation was a strong posture dependent difference in relative muscle recruitment, with marked attenuation of evoked responses in the standing position compared with supine across muscle groups, independent of stimulation intensity, pulse width, and electrode configuration. Proximal muscles, including HS and VM, tended to reach recruitment plateaus at lower stimulation intensities, whereas distal muscles, MG and TA, more often exhibited graded increases with increasing stimulation.

These findings are consistent with prior work demonstrating that body position can influence neural recruitment during spinal stimulation. In lumbar tSCS, Danner et al. showed that posture altered the neural structures recruited and modified evoked motor responses in neurologically intact participants supporting the concept that stimulation effects depend not only on electrode placement and stimulation parameters but also on the biomechanical and sensory state of the participant [14]. Similarly, Sayenko et al. demonstrated differences in MEPs of lower limb musculature in persons with SCI as a result of postural changes and position of rostro-caudal stimulation [21]. The present case report extends this concept to pSCES by demonstrating that posture-dependent attenuation of evoked motor responses can persist across stimulation intensity, electrode configuration, and pulse-width conditions during pSCES mapping.

The physiological origin of the recorded responses should be interpreted with caution. Based on the posterior epidural location of the leads and their anatomical position over the lumbosacral enlargement, the observed EMG responses are consistent with pSCES-evoked activation of spinal sensorimotor circuitry, likely involving recruitment of posterior root afferents and reflex-mediated pathways, including posterior root-muscle reflexes as previously described [22]. This interpretation is further supported by prior work showing that epidural spinal stimulation can evoke motor responses through activation of lumbosacral spinal networks [7–11]. However, because response latency was not analyzed and paired-pulse stimulation was not performed, the present study cannot definitively distinguish reflex-mediated components from possible direct activation of motor pathways. Therefore, the findings should be interpreted as posture-dependent modulation of pSCES-evoked motor responses rather than definitive evidence of a specific neural pathway.

Another major finding was that the HS muscle consistently demonstrated the greatest magnitude of activation across experimental conditions in both postures. According to prior myotomal mapping, innervation of HS musculature primarily involves the L5 and S1 spinal nerve roots, which are influenced by the lateral corticospinal tract (LCST), an upper motor neuron pathway originating in the precentral gyrus and terminating in the anterior horn of the spinal cord [23]. The LCST has been previously described to have somatotopic organization, with neurons of cervical segments positioned medially and lumbosacral segments positioned laterally [24]. Taking this into consideration, preferential activation of HS muscle in both participants regardless of posture may suggest that lead placement relative to key neuroanatomical structures could lead to precise targeted muscle activation, supporting previous findings of SCS position-dependent muscle recruitment [25].

Additionally, despite testing various combinations of configurations, pulse widths, and stimulation intensities, the alteration of pSCES parameters could not overcome postural dependent decreases in muscle recruitment. Despite utilizing both wide field and narrow field configurations, muscle motor evoked responses were attenuated by altering posture. Similarly, while increasing pulse width further expanded overall recruitment, most notably in the supine posture, standing responses remained comparatively limited. Along the same lines, alterations in stimulation intensity generally resulted in increased muscle recruitment with varying involvement of the proximal versus distal muscles, but again could not overcome postural differences. These findings suggest that fine tuning pSCES parameters may improve muscle recruitment to an extent. However, increasing pulse width and stimulation intensity did not eliminate posture-dependent differences in muscle recruitment after SCI. Although the parameters tested were not exhaustive, consideration of parameter optimization may be needed to mitigate the differences in muscle recruitment to enhance functional activities with changes in posture.

These findings have several potential implications for applications of pSCES in SCI. Prior work has described decreased amplitude of the soleus H-reflex in the standing position of neurologically intact individuals indicating that presynaptic inhibition of Ia afferents leads to decreased motoneuron activation [26–30]. The same neural pathway has been also shown to be activated by SCES and tSCS with resultant muscle recruitment [22]. Furthermore, this reflex modulation remains intact in persons with SCI below the level of injury [29]. Given the persistence of this reflex modulation, enhanced activation of residual spinal sensory and proprioceptive circuitry in the standing position may contribute to presynaptic inhibition of Ia afferents, thereby reducing effective motoneuron recruitment during upright posture by pSCES [30]. We have recently noted that eliciting H-reflex response in a standing posture is rather challenging following hybrid exoskeletal assisted walking and pSCES in persons with SCI [31]. Within the same Ia afferent-motoneuron reflex circuitry, increased afferent input from proprioceptive signals and muscle loading in the standing position compared to supine may also lead to decreased motor activation via synaptic depression of Ia afferent fibers – a mechanism that demonstrated previously following repetitive stimulation that led to the depression of the H-reflex [32]. These two mechanisms of reflex modulation that are based on increasing proprioceptive and sensory inputs, in relation to altering posture, may explain the attenuation of MEPs. In addition, posture related alterations in CSF distribution and dural sac geometry may modify pSCES current propagation within the spinal canal and attenuate stimulation efficacy in standing [33, 34]. The present findings are consistent with other research studies that indicated that dorsal CSF may attenuate the propagation of the current to activate the target neural circuitries [17, 18]. This represents an important consideration during spinal mapping, which is a critical step in translating electrical stimulation into functional motor output required for activities such as standing [12]. The preliminary findings suggest that needs to explore a potential method for maintaining the proximity of pSCES leads to the corresponding spinal cord circuitries. Wearing a lumbosacral belt may increase the intrabdominal pressure [35] and may reduce the distance between pSCES electrodes and the spinal cord in standing posture. Because descending pathway integrity was not directly assessed in the present study, pathway-specific mechanisms underlying the observed attenuation should be interpreted cautiously and evaluated in future studies.

## Limitations

Several limitations of the present analysis should be acknowledged. First, this case report included only two participants with chronic motor complete SCI, which limited generalizability and may necessitate cautious interpretation when applying these findings to individuals with incomplete SCI or different neurological classifications. The sample size reflects the pilot nature of this initial posture-specific pSCES mapping analysis within the ongoing parent trial. Because the mapping protocol was still being developed and refined, and because posture-specific mapping was not the primary efficacy endpoint of the parent trial, the present report focused on detailed characterization of the initial participants rather than broad efficacy testing. Future studies with larger samples are warranted to confirm generalizability of these posture-dependent recruitment patterns. Further, recruitment curves were generated using 1 mA stimulation increments, which may have reduced sensitivity for detecting subtle posture-dependent shifts in muscle recruitment compared with finer increments of 0.1 mA. In addition, normalization procedures were performed within-session and relative to maximal responses obtained across tested configurations, which may influence comparisons of muscle recruitment between supine and upright postures. Surface EMG recordings are also inherently susceptible to variability related to electrode placement, soft tissue characteristics, and postural changes in muscle size, which may have contributed to variability across measured amplitudes and different stimulation configurations. In addition, response latency was not analyzed and paired-pulse stimulation was not performed during these mapping sessions; therefore, the present study could not directly verify the relative contribution of reflex-mediated versus direct motor components to the recorded EMG responses. Future studies incorporating latency-based response classification and paired-pulse paradigms may help further characterize the physiological origin of pSCES-evoked responses across postural conditions. Finally, inter-individual differences of spinal cord anatomy, residual circuitry, and responsiveness to pSCES may have influenced the observed posture-dependent recruitment patterns.

## Conclusions

The findings demonstrated that body posture is a dominant determinant of pSCES-evoked motor recruitment in persons with motor complete SCI. Transitioning from supine to standing demonstrated consistent attenuation of both raw and normalized EMG responses across stimulation intensities, pulse widths, and pSCES electrode configurations, despite preservation of intensity-dependent recruitment curve patterns. Proximal musculature demonstrated preferential recruitment, whereas distal muscle activation remained limited in the upright posture, even with wider field configuration and longer pulse durations. These findings indicated posture-dependent responses to pSCES and emphasize the importance of accounting for posture during pSCES mapping. Parameter optimization with particular consideration of increasing pulse duration may facilitate muscle recruitment to increase overall muscle involvement and to mitigate postural-dependent differences. Accounting for posture-dependent modulation of stimulation efficacy may assist researchers and clinicians in refining stimulation strategies during configuration selection for functional tasks such as standing. Ultimately, integrating posture-specific recruitment characteristics may serve as a step toward developing adaptive stimulation frameworks that improve functional outcomes following pSCES implantation in individuals with SCI.

## Data Availability

Data will be shared by the lead contact author [Ashraf Gorgey (ashraf.gorgey@va.gov)] upon request and after obtaining necessary approval from the local research office.
